# Induction of oxidative stress, apoptosis and DNA damage by koumine in *Tetrahymena thermophila*

**DOI:** 10.1371/journal.pone.0212231

**Published:** 2019-02-12

**Authors:** Qiao Ye, Chaonan Zhang, Zhenlu Wang, Yongyong Feng, Aiguo Zhou, Shaolin Xie, Qiong Xiang, Enfeng Song, Jixing Zou

**Affiliations:** 1 Healthy Aquaculture Laboratory, College of Marine Sciences, South China Agricultural University, Guangzhou, Guangdong, China; 2 Joint Laboratory of Guangdong Province and Hong Kong Region on Marine Bioresource Conservation and Exploitation, College of Marine Sciences, South China Agricultural University, Guangzhou, Guangdong, China; 3 Department of Traditional Chinese Medicine, Renmin Hospital of Wuhan University, Wuhan, Hubei, China; Nanjing University, CHINA

## Abstract

Koumine is a component of the Chinese medicinal herb *Gelsemium elegans* and is toxic to vertebrates. We used the ciliate *Tetrahymena thermophila* as a model to evaluate the toxic effects of this indole alkaloid in eukaryotic microorganisms. Koumine inhibited *T*. *thermophila* growth and viability in a dose-dependent manner. Moreover, this drug produced oxidative stress in *T*. *thermophila* cells and expressions of antioxidant enzymes were significantly elevated at high koumine levels (*p* < 0.05). Koumine also caused significant levels of apoptosis (*p* < 0.05) and induced DNA damage in a dose-dependent manner. Mitophagic vacuoles were present in cells indicating induction of autophagy by this drug. Expression of *ATG7*, *MTT2/4*, *CYP1* and *HSP70* as well as the MAP kinase pathway gene *MPK1* and *MPK3* were significantly altered after exposed to koumine. This study represents a preliminary toxicological evaluation of koumine in the single celled eukaryote *T*. *thermophila*.

## Introduction

*Gelsemium elegans* Benth is a genus in the family Loganiaceae that are widely distributed in China and Southeast Asia, and it is highly toxic to mammals [1] and its poisoning is usually through inadvertent ingestion [2]. However, *G*. *elegans* Benth is used as a Chinese folk medicine for treatment of tumors [3], neuropathic pain, inflammation [4], rheumatic arthritis [5], and anxiety [6, 7]. *G*. *elegans* contains a variety of active constituents including alkaloids, the iridoid monoterpenes and triterpenes. Over a hundred alkaloids have been identified from *G*. *elegans* and predominant indole alkaloids include koumine, gelsemine, gelsemicine, etc [8, 9]. The indole alkaloid koumine is the most abundant of these alkaloids that are structurally similar but are diverse in pharmacological action and toxicity, and its biological effects are poorly described. Studies have shown koumine may enhances spinal cord 3α-hydroxysteroid oxidoreductase expression and activity in a rat model of neuropathic pain and that the reduction in neuropathic pain may be associated with the upregulation of allopregnanolone in the spinal cord [10, 11]. The subjects of these studies were limited to rats and mouse. The responses of different species to toxicity vary greatly, especially in vertebrates and non-vertebrates. Therefore, there has been an increasing interest to investigate the toxicity of koumine in eukaryotic microorganisms.

Furthermore, pathogenetic eukaryotic microorganism, such as *Echinococcus multilocularis*, *Ichthyophthirius multifiliis* and *Vorticella*, caused potential aquatic ecological and human health risks [12, 13]. *T*. *thermophila* can be used as a research model for these pathogenic microorganisms. *T*. *thermophila* is a unicellular ciliate distributed in freshwater waters around the world that has been extensively used for toxicology studies [14]. *T*. *thermophila* is sensitive to diverse pollutants and toxic agents including heavy metals [15, 16, 17] and shares a higher degree of functional conservation with human genes than other microbial model eukaryotic microorganisms [18]. *T*. *thermophila* grows rapidly to a high cell density in a variety of media and its genome has been sequenced [19]. As described above, *T*. *thermophila* makes our toxicity study more convenient. Therefore, *T*. *thermophila* is an ideal biological model to study koumine toxicity.

To perform toxicity investigations, it is important to wisely choose the indices at various biological levels. Studies have shown that the reproduction at the population level can change antioxidant capacities and result in oxidative stress [20, 21]. The antioxidant indices including SOD, POD, CAT, MDA and GSH-PX were usually considered in assessing effects of toxicants [22]. These indices were activated to counteract oxidative stress and maintaining organism stability. The changes of antioxidant capacities by chemical exposures (e.g., melamine) resulted in oxidative stress that altered apoptosis rate and DNA damage level [23]. The activation of apoptosis is usually through caspases 3 pathway, and caspases 8 and 9 amplify the cascade effect [24]. The toxicity of chemical compound can be roughly understood by the above-mentioned indicators. Therefore, the effects on growth, oxidative stress, DNA damage and apoptosis in *T*. *thermophila* would help understanding the toxicity of koumine.

It is also important to study the expression of oxidative stress—related genes in toxicological studies. Thus, we chose several oxidative stress-related genes to study the genotoxicity of koumine to *T*. *thermophila*. Metallothioneins (MTTs) are widely distributed among organisms and are involved in oxidative stress [25], and cytochrome P450, family 1 (*CYP1*) is also increased by oxidative stress [26]. The heat shock protein 70 (*HSP70*) is a family of conserved and ubiquitously expressed heat shock proteins that protect cells from stress [27]. Mitogen-activated Protein Kinase 1 and 3 (*MPK1* and *MPK3)* are critical components of MAP kinase signaling and their expression is related to oxidative stress [28, 29]. Autophagy 7 (*ATG7*) gene plays an important role in autophagy signal transduction and oxidative stress [30]. Changes in the expression of these genes can be used as part of toxicity assessment.

To the best of our knowledge, koumine toxicity for eukaryotic microorganisms including *T*. *thermophila* has not been reported. In this study, we used the *T*. *thermophila* model to investigate toxicity of koumine and assessed its growth inhibitory, oxidative stress, DNA damage and apoptosis effects. This study provides preliminary reference data for the toxicity of koumine to eukaryotic microorganisms.

## Materials and methods

### Processing of koumine

Koumine was purchased from Chengdu Mansite Bio-Technology (Chengdu, China). Since koumine decomposes into dimethyl sulfoxide at 60°C, the compound was exposed to ultraviolet germicidal irradiation for 1 h to inactivate any microorganisms present.

### Culture of *T*. *thermophila*

*T*. *thermophila* strain B2086 was generously provided by Professor Miao (Chinese Academy of Sciences, Wuhan, China). *T*. *thermophila* was inoculated into Super proteose peptone (SPP) medium, which consists of 2% proteose peptone, 0.1% yeast extract, 0.2% glucose and 0.003% ferric citrate, and was cultured at 30°C [31]. For use in experimental procedures, the cells were centrifuged at 3500 rpm for 15 min and suspended in phosphate buffered saline (PBS, pH 7.4).

### Exposure of koumine

*T*. *thermophila* cells were cultured in SPP in test tubes containing 5 mL of medium and koumine was added at 0, 0.05, 0.1, 0.2, 0.4 and 0.8 mg/mL. 0 mg/mL koumine was used as blank control and 0.1% DMSO was used as negative control (NC). Penicillin G (100 units/mL), streptomycin (100 μg/mL) and amphotericin B (0.02 μg/mL) were then added to each tube of medium to prevent bacterial and fungal contamination. Cells were inoculated to a final density of 6250 cells/mL and tubes were incubated for 72 h at 30°C with constant shaking at 135 rpm [31]. Cells were counted using an automated cell counter (Countstar, Shanghai, China). Cell viability was determined at 24 h using a Cell Counting Kit-8 (CCK-8) (Nanjing Jiancheng, Nanjing, China) according to the manufacturer’s instructions [32]. The oxidative stress, apoptosis, DNA damage, ultrastructural changes and gene expression effects was determined after *T*. *thermophila* exposed to various levels koumine at 24 h. The specific experimental process was shown below.

### Antioxidant activity assays

After 24 h culture, the activities of superoxide dismutase (SOD), catalase (CAT), peroxidase (POD), glutathione peroxidase (GSH-PX), caspases (caspases 3, 8, 9) and malonaldehyde (MDA) contents were measured using commercial assay kits (Nanjing Jiancheng Biotechnology, Nanjing, China) according to the manufacturer’s protocols [33]. Oxidative stress was evaluated by measuring intracellular reactive oxygen species (ROS) using the fluorescent dye DCFH-DA. Cells were collected by centrifugation and suspended in culture medium and transferred to 96-well plates. DCFH-DA (10 μM) was added and the plates were incubated for 40 min at 37°C. DCFH-DA was removed by centrifugation and the cells were washed three times with culture medium and fluorescence was measured using a Multiskan microplate reader (Thermo, Pittsburg, PA, USA) with 485nm excitation and 525 nm emission wavelengths.

### Examination of apoptosis using flow cytometry

Cells are prepared as previously described and apoptosis was examined using flow cytometry [34]. Briefly, fluorescein isothiocyanate (FITC) -Annexin V was added to *T*. *thermophila* cells and incubated for 10 minutes in an ice bath in the dark. Cells were washed 2 times with PBS and analyzed using a Beckman-Coulter EPICS XL flow cytometer (Beckman, Richmond, CA, USA) with a 488 nm argon ion laser light source. A total of 10, 000 cells were obtained and examined per sample. Data were analyzed using CellQuest software provided with the instrument.

### Comet assay and ultrastructural analysis

The comet assay was applied to detect the DNA damage of *T*. *thermophila* exposed to koumine at 24 h using a commercial kit (Nanjing Jiancheng) according to the manufacturer’s instructions. The basic steps were as follows: suspensions of *T*. *thermophila* cells were prepared; and the cells were then embedded in agarose on microscope slides; cells were split to release the DNA; single-stranded DNA was obtained using an alkaline solution and subjected to electrophoresis under alkaline conditions; the DNA was fluorescently stained by ethidium bromide (EB), and the comets were visualized; the number of comet cells was counted, and the oliver tail moment (OTM) was measured. The cells were observed and imaged under a fluorescence microscope and the pictures were analyzed using commercial software (CASP 1.2.3 beta 1).

Transmission electron microscopy (TEM) imaging was performed by ServiceBio (Wuhan, China) [35]. Briefly, *T*. *thermophila* cells were collected and fixed in 2.5% glutaraldehyde. After that, cells were scraped, pelleted, dehydrated, infiltrated and embedded, and then ultrathin sections were obtained and stained, Hitachi HT7700 TEM was used for imaging of *T*. *thermophila* ultrastructure.

### Gene expression analysis

Total RNA was isolated from *T*. *thermophila* using an RNAiso Plus kit (Takara, Dalian, China) according to the manufacturer’s protocol. The OD260/OD280 ratio was measured using UV spectroscopy and RNA integrity was evaluated by 2% agarose gel electrophoresis and reverse transcribed using Takara reverse transcription kit (Takara, Dalian, China) according to the manufacturer’s instructions. The cDNA were used as templates for the gene amplification in a fluorescence quantitative PCR assay.

The mRNA sequence of the *T*. *thermophila MTT2/4* (Metallothionein 2, TTHERM_00433520 and TTHERM_00433530), *CYP1* (Cytochrome P450, family 1, TTHERM_00881440), *HSP70* (Heat Shock Protein 70, TTHERM_00105110), *MPK1* (Mitogen-activated Protein Kinase 1, TTHERM_00469230), *MPK3* (Mitogen-activated Protein Kinase 3, TTHERM_00660130), *ATG7* (Autophagy 7, TTHERM_00138370) genes and 18S ribosomal RNA (HE820726.1) were obtained from Tetrahymena Genome Database (http://ciliate.org/index.php/home/welcome). Specific primers suitable for quantitative real-time polymerase chain reaction (RT-qPCR) were designed using Primer Premier 5.0 software (Table A in S1 File). Relative gene expression levels were normalized against the expression level of 18S rRNA. Standard curves of primers using in RT-qPCR are provided in Supporting information (Figs A, B, C, D, E, F, G in S1 File). RT-qPCR was performed using a standard SYBR Premix Ex Taq II (Takara, Dalian, China) on a Biorad CFX96 Real-Time PCR Detection System (Hercules, CA, USA) according to the manufacturer's protocol. Thermal cycling conditions were 95°C for 2 min, followed by 39 cycles of 95°C for 15 s, 60°C for 30 s, 72°C for 30 s and a final cycle of 72°C for 7 min. Melting curves were performed from 60 to 90°C. Relative gene expression was calculated relative to the quantity of 18S rRNA present.

### Statistical analysis

Experiments were carried out at least in triplicate. Data from cell viability assays, DNA damage and antioxidant index tests were analyzed using SPSS statistical package version 19.0. Differences were calculated by analysis of variance (ANOVA). Real-time PCR data were analyzed using the 2^-ΔΔCt^ method and differences between the groups were analyzed using the t-test. A value of *p* < 0.05 was considered statistically significant. All values were expressed as the mean ± standard deviation (SD).

The half-maximal effective concentration (EC50) of koumine was calculated using cell viability data and all experiments contained three biological replicates. For the purpose of EC50 calculation, the data were fitted to the classical sigmoidal four parameter dose-response model:

$$y=b+\frac{a-b}{1+10^{\wedge }(LogEC50-x)h}$$

where *y* is the response, *b* represents the minimum of the response, *a* represents the maximum of the response, *h* is the shape parameter, *x* is the logarithm of inhibitor concentration. EC50 values were calculated with their 95% confidence interval from the data obtained using the software package GraphPad Prism 5.0.

## Results

### Effects on cells growth and viabilities

**Fig 1:**
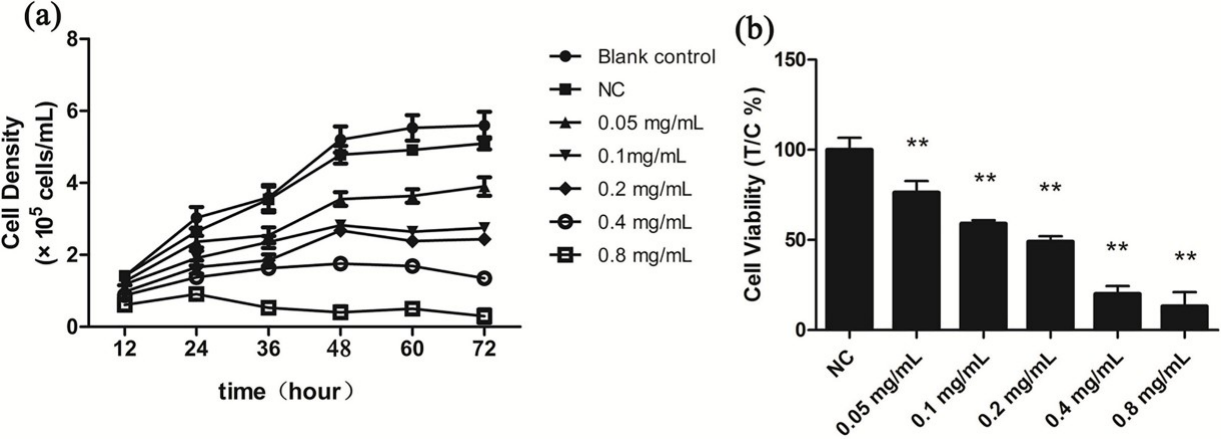
The effect of koumine on *T*. *thermophila* growth after various levels exposure. (a) *T*. *thermophila* growth in the presence of koumine for 72h. (b) *T*. *thermophila* viability after koumine exposure for 24h. Data are expressed as mean ± SD (n = 3). * indicates significant differences (*P* < 0.05), ** indicates highly significant differences (*P* < 0.01). Blank control, no drug addition. NC, Negative control (0.1% DMSO). All treatment groups were compared to the NC group.

We used growth of *T*. *thermophila* in the presence and absence of koumine to develop an initial indication of its toxic effects under ideal growth conditions. Cell densities declined as the concentration of koumine increased in a concentration-dependent manner (Fig 1A). Compared with NC, all koumine doses from 0.05 to 0.8 mg/mL generated statistically significant declines in growth (Table B in S1 File). In addition, cell viability of all koumine treatment groups decreased significantly compared with NC (*P* < 0.01) (Fig 1B). These reductions in viability were dose-dependent and were most significant at 0.4 and 0.8 mg/mL koumine. We also determined that the EC50 at 24 h was 0.23±0.04 mg/mL indicating a low tolerance for the drug. At a concentration of 0.05 mg/mL we also noted significant differences compared to NC although the effect was not as dramatic as for the higher levels.

### Effects on oxidative stress and apoptosis

**Fig 2:**
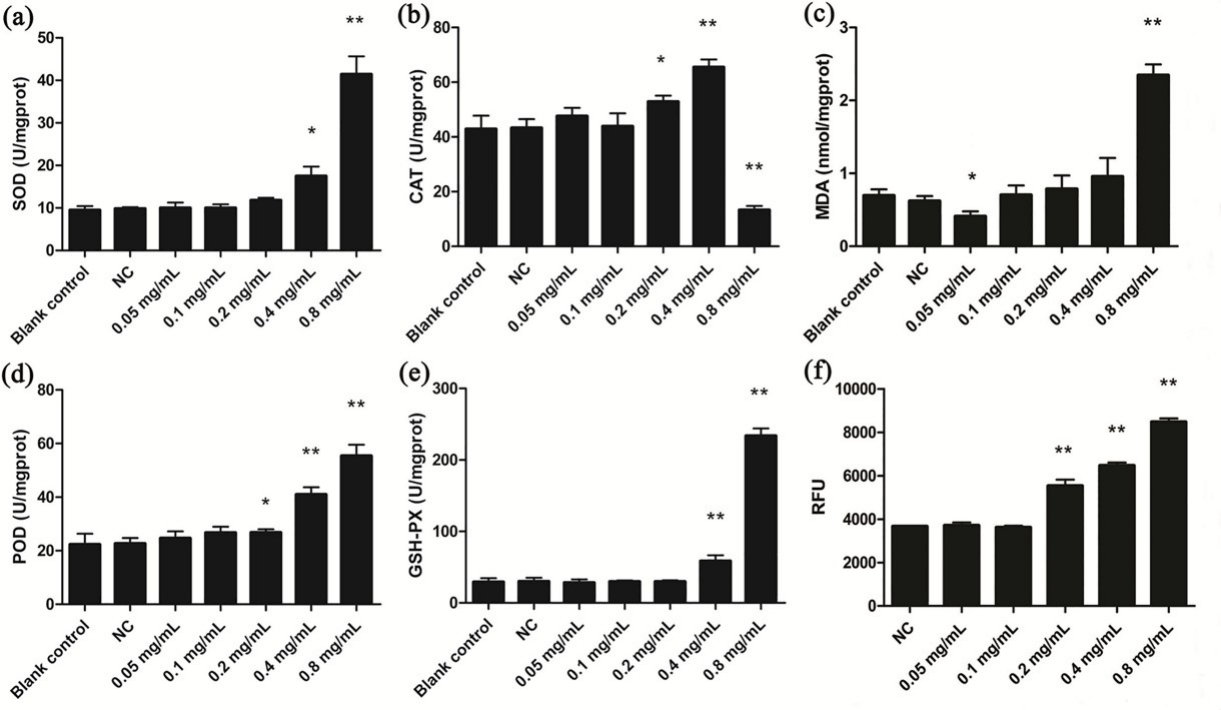
Antioxidant indices for *T*. *thermophila* exposure to koumine. (a) SOD (b) CAT (c) MDA (d) POD (e) GSH-PX (f) ROS, results were expressed as relative fluorescence unit (RFU) values. Results were expressed as mean ± SD (n = 3). * indicates significant differences (*P* < 0.05), ** indicates highly significant differences (*P* < 0.01). All treatment groups were compared to the NC group.

We next examined whether koumine addition altered antioxidant enzyme and MDA levels. SOD and GSH-PX activities in cells exposed to 0.4 and 0.8 mg/mL koumine were significantly increased (*p* < 0.05). However, no significant changes of SOD and GSH-PX were observed in the other treatment groups (Fig 2A and 2E). CAT and POD activities at 0.2, 0.4 and 0.8 mg/mL koumine were also increased significantly (*p* < 0.05). Interestingly, CAT levels significantly declined at 0.8 mg/mL of koumine (*p* < 0.05) (Fig 2B and 2D).

The cellular MDA content increased to extremely significant levels at 0.8 mg/mL koumine (*p* < 0.01) and significantly decreased at 0.05 mg/mL koumine compared to NC (Fig 2C). In addition, ROS levels were extremely significantly increased at 0.4 and 0.8 mg/mL koumine (Fig 2F). Overall, the 4 antioxidant enzymes and MDA were all changed significantly at lower koumine levels (Fig 2). This indicated that koumine caused oxidative stress in *T*. *thermophila* cells.

**Fig 3:**
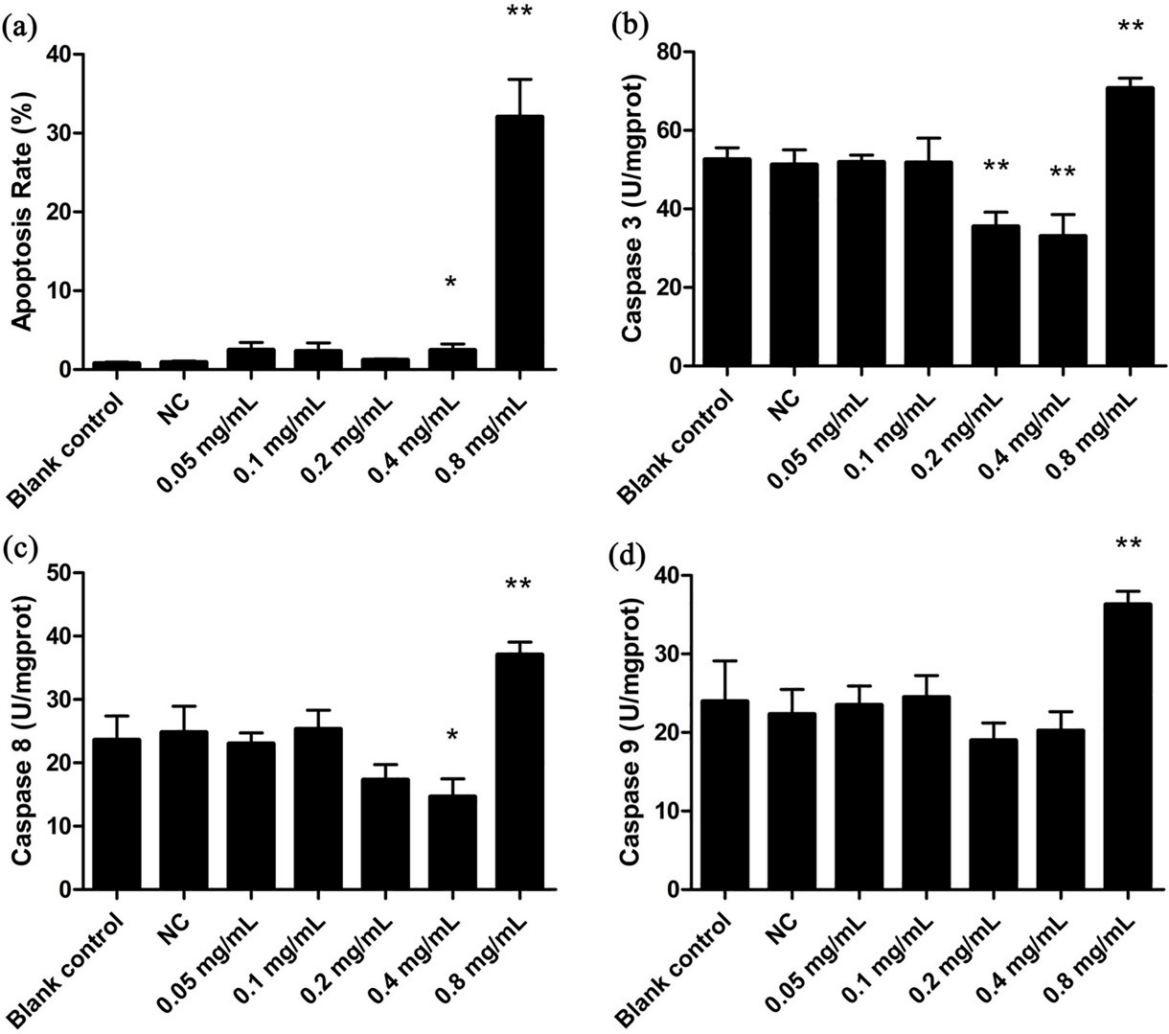
Apoptosis of *T*. *thermophila* after koumine exposure. (a) Apoptosis rate and activities of (b) Caspase 3 (c) Caspase 8 and (d) Caspase 9. Data were expressed as mean ± SD (n = 3). * indicates significant differences (*P* < 0.05), ** indicates highly significant differences (*P* < 0.01). All treatment groups were compared to the NC group.

**Fig 4:**
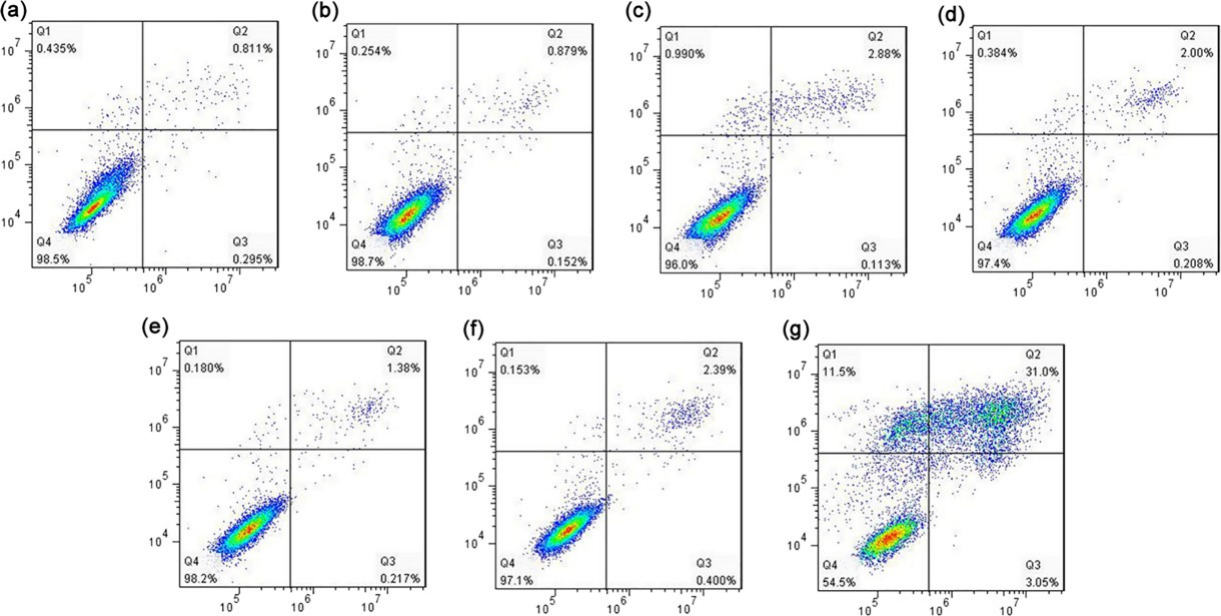
Flow cytometry analysis of apoptosis in *T*. *thermophila*. (a) Blank control; (b) negative control (NC) and koumine added at (c) 0.05 mg/mL; (d) 0.1 mg/mL; (e) 0.2 mg/mL; (f) 0.4 mg/mL and (g) 0.8 mg/mL. Q1: necrotic cells; Q2: late stage apoptotic cells; Q3: early stage apoptotic cells; Q4: living cells.

When we examined whether koumine stress also results in apoptosis, the apoptosis rates in the blank control and the NC, 0.05, 0.1, 0.2 and 0.4 mg/mL groups were all <5%. This indicated that that apoptosis was a low probability occurrence. However, at 0.8 mg/mL koumine approximately 31% of cells underwent apoptosis (Fig 3A and Fig 4). Caspase 3 activity in cells exposed to 0.2 and 0.4 mg/mL koumine were significantly decreased (*p* < 0.01) compared to NC (Fig 3B) and caspase 8 levels were significantly decreased at 0.4 mg/mL koumine (*p* < 0.05) (Fig 3C). In contrast, caspase 9 levels significantly increased at 0.8 mg/mL (*p* < 0.05) (Fig 3D). However, these 3 caspases were all significantly elevated at 0.8 mg/mL (Fig 3B, 3C and 3D).Therefore koumine at 0.8 mg/mL was apoptotic to *T*. *thermophila*.

### DNA and ultrastructural damaging effects of koumine for *T*. *thermophila*

**Fig 5:**
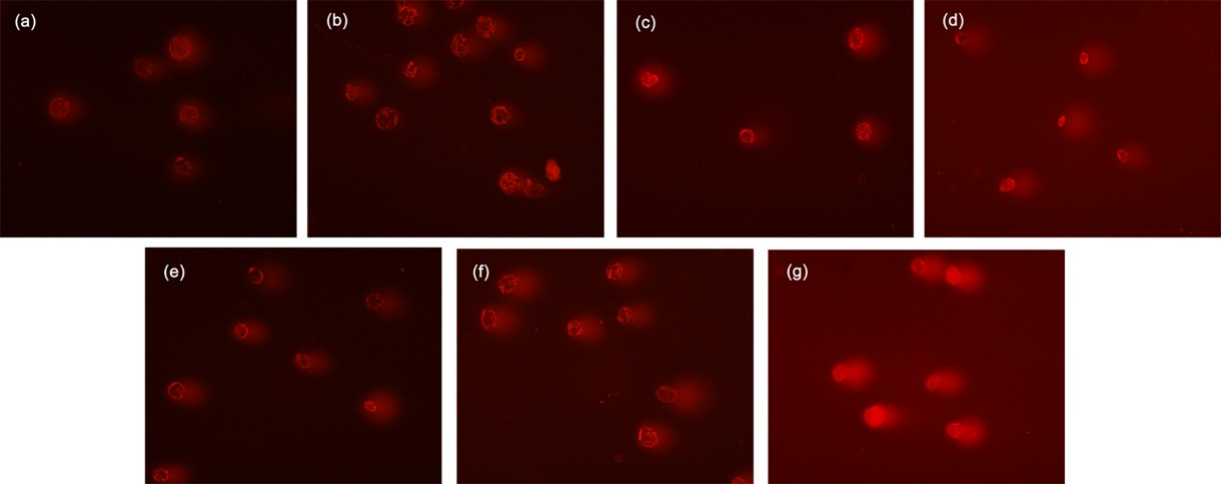
Comet assay of *T*. *thermophila* cells after koumine exposure. (a) Blank control; (b) negative control (NC) and koumine added at (c) 0.05 mg/mL; (d) 0.1 mg/mL; (e) 0.2 mg/mL; (f) 0.4 mg/mL; (g) 0.8 mg/mL. Magnification was 20×.

**Fig 6:**
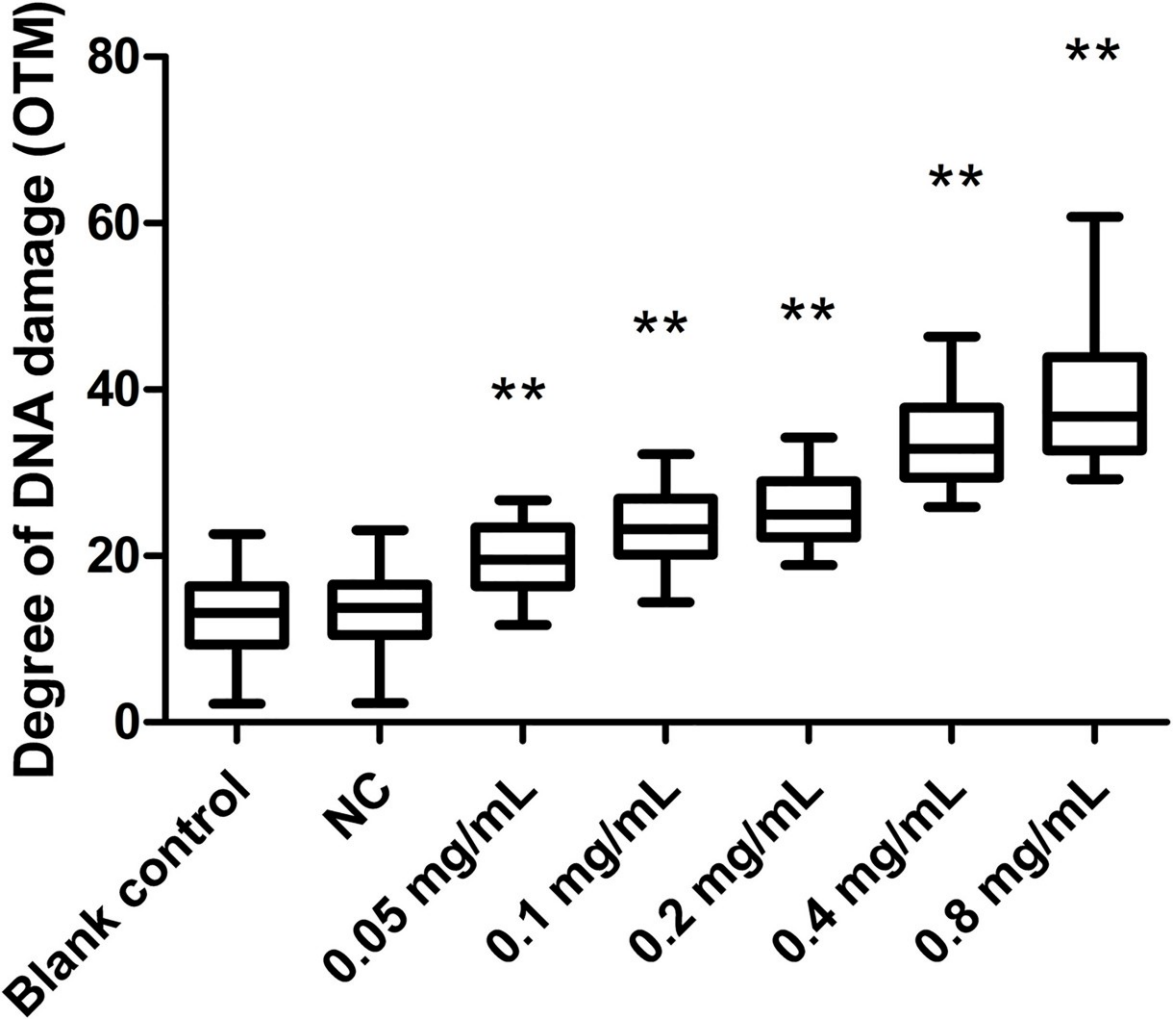
DNA damage in *T*. *thermophila* exposed to increasing concentrations of koumine for 24 h. Olive tail moment (OTM) values were measured using Comet Assay Software (CASP 1.2.3 beta 1). Data were expressed as mean ± SD (n = 3). * indicates significant differences (*P* < 0.05), ** indicates highly significant differences (*P* < 0.01). All the treatment groups were compared to the NC group.

We assessed DNA damage in *T*. *thermophila* using the comet assay and koumine caused a dose-dependent DNA damage response that was severe at 0.8 mg/mL (Fig 5). The olive tail moment (OTM) values were significantly increased in all koumine treatment groups (*p* < 0.05). At 0.8 mg/mL koumine, OTM values were 1.8 fold higher than for the 0.05 mg/mL group (Fig 6). In addition, koumine caused mid-level DNA damage (40–60%) at dosages of 0.4 and 0.8 mg/mL (Table 1). These results indicated that koumine produced significant DNA damage to *T*. *thermophila*.

**Table 1:** Cells in different DNA damage classes measured in *T*. *thermophila* exposed to koumine.

Damage classes	Blank control	NC	koumine (mg/mL)				
			0.05	0.1	0.2	0.4	0.8
Min	110	113	89	63	48	11	4
Low	29	26	38	50	56	21	12
Mid	4	3	17	24	37	70	66
High	1	0	1	6	6	31	51
Extreme	0	0	0	0	0	2	12

Min = minimal damage <20% DNA in the comet tail; Low = low damage 20–40% DNA in the comet tail; Mid = mid damage 40–60% DNA in the comet tail; High = high damage 60–80% DNA in the comet tail and Extreme = extreme damage >80% DNA in the comet tail.

**Fig 7:**
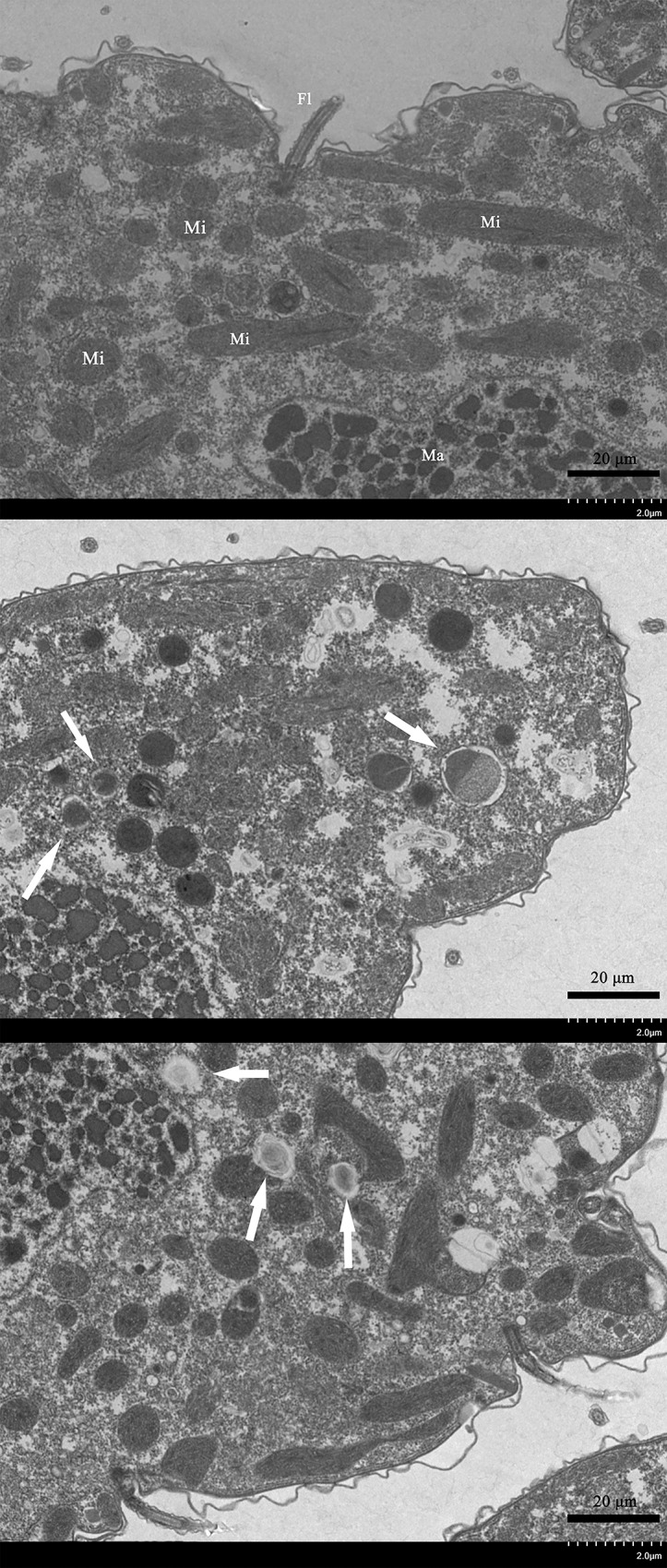
Ultrastructural details of *T*. *thermophila* cells exposed to increasing concentrations of koumine. (a) NC group. Macronucleus (Ma), Mitochondria (Mi), Flagellum (Fl); *T*. *thermophila* cells exposed to koumine at (b) 0.4 mg/mL and (c) 0.8 mg/mL. Arrows, mitophagic vacuoles.

In addition, we found that koumine also produced ultrastructural damage to *T*. *thermophila* cells. At 0.4 and 0.8 mg/mL, we observed numerous mitophagic vacuoles (compare Fig 7A with 7B and 7C). These most likely were the reason for cell death in the koumine treatment groups.

### Oxidative stress related genes expression in *T*. *thermophila*

**Fig 8:**
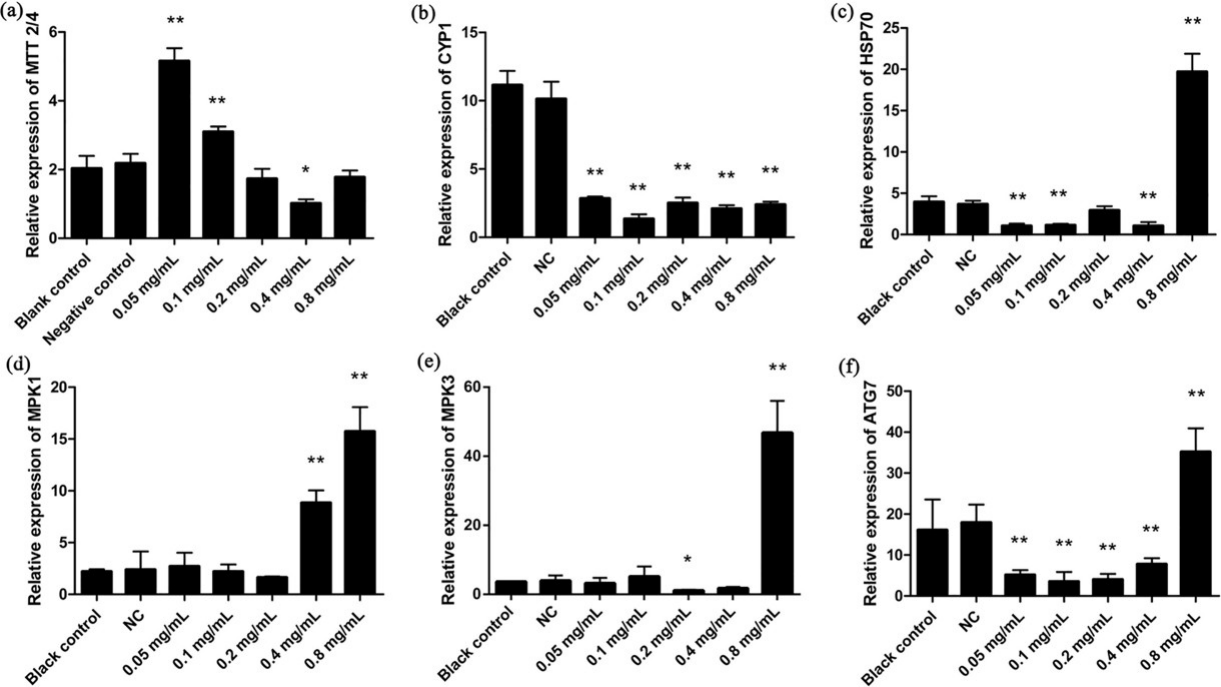
Oxidative stress related genes in *T*. *thermophila* after koumine treatment (24 h). (a) *MTT2/4*; (b) *CYP1*; (c) *HSP70*; (d) *MPK1*; (e) *MPK3* g; (f) *ATG7*. Results were normalized against 18S rRNA. Data were expressed as mean ± SD (n = 3). * indicates significant differences (*P* < 0.05), ** indicates highly significant differences (*P* < 0.01). All the treatment groups were compared to the NC group.

We used expression analysis of oxidative stress responsive genes as an additional assay for oxidative stress. Expression of metallothionein (MT) *MTT2/4* was increased significantly at 0.05 and 0.1 mg/mL koumine (*p* < 0.01) although it was significantly reduced at the 0.4 mg/mL level (*p* < 0.05) (Fig 8A). Cytochrome P450 (*CYP1*) expression significantly decreased at all dosage levels compared to NC (*p* < 0.01) (Fig 8B). *HSP70* significantly decreased in all treatment groups with the exception of the 0.8 mg/mL group compared to NC (*p* < 0.01) (Fig 8C). The expression levels of MAP kinase pathway members *MPK1* and *MPK3* and the autophagy protein *ATG7* were all significantly increased at 0.8 mg/mL koumine (Fig 8D, 8E and 8F).

## Discussion

At present, koumine research mainly focuses on neuropathic pain and inflammation and the main object of studies were vertebrates [10]. Eukaryotic microorganisms and vertebrates respond differently to toxic substances. Furthermore, koumine is not a common toxic substance and this is most likely the reason it has received only little attention to eukaryotic microorganisms. Our research fills the gap in the toxicity of koumine to eukaryotic microorganisms. *T*. *thermophila*, widely present in water environment, is a proven and useful tool for toxicological studies [36, 37] and koumine toxicity to this organism is an important reference value for other pathogenic eukaryotic microorganisms [13]. In the present study, we found that *T*. *thermophila* growth and viability were negatively correlated with koumine especially at the higher dose levels. These koumine exposure also led to increased ROS production that led to oxidative stress. To maintain homeostasis, antioxidant defense systems including SOD, POD, CAT and GSH-PX were activated in order to counteract oxidative stress and oxidant damage [22]. MDA levels were also increased and were indicative of lipid peroxidation by koumine reflecting the overall level of oxidative stress [38]. An increase in antioxidant enzyme activity is directly linked to ROS production [39]. Only under extreme conditions were these antioxidants synthesized (at 0.4 and 0.8 mg/mL koumine) and the cell population was ~90% dead. These enzymatic activities were produced in an attempt to overcome severe oxidative stress caused by koumine. Excessive oxidative stress may also induce apoptosis in cells [40, 41]. In the present study, we found that the apoptosis rate was significantly elevated at 0.8 mg/mL and caspase 3, 8 and 9 levels at 0.8 mg/mL treatment groups were significantly elevated. In apoptotic pathways, the activation of caspase cascades usually triggered by caspases 3. Caspase 3 initiates apoptotic damage and caspases 8 and 9 amplify the cascade [24]. Based on the current data, high koumine level induce apoptosis in *T*. *thermophila* cells that is mediated by activation of caspases 3, 8 and 9. The caspases 3 also associated with DNA damage [42]. We found that the levels of DNA damage level(OTM value) increased with the increase of caspase expression at 0.4 and 0.8 mg/mL koumine.

Oxidative stress can cause DNA damage in tissues and cells [43, 44, 45]. We used the comet assay to evaluate DNA damage and the OTM is an important index for evaluating DNA damage in the comet assay [46]. The comet assay results indicated that koumine induced DNA damage in *T*. *thermophila* in a dose-dependent manner. The serious DNA damage and severe oxidative stress also induced disturbed mitochondrial integrity and function [47, 48, 49]. We identified mitophagic vacuoles in cells exposed to koumine at 0.4 and 0.8 mg/mL indicating this organelle is a primary koumine target. DNA damage and mitophagic vacuoles may be caused by oxidative stress. DNA damage levels at 0.8 mg/ml koumine were serious and present in about 89% of cells exposed to the drug. From the above indicators, we can have a preliminary understanding of the toxicity of koumine to *T*. *thermophila*.

The expression profile of oxidative stress-related genes is equally important in toxicity assessment. Metallothioneins are widely distributed in organisms and are involved in a wide variety of biological processes including inhibition of oxidative stress [50]. MTTs expression can be induced by oxidative stress and toxic substances [25]. We selected *MTT2/4* genes to study the changes of expression level exposed to various koumine concentrations. *MTT2/4* expression reached a peak at 0.05 mg/mL koumine while other oxidative stress-related genes (*CYP1*, *HSP70*, *MPK1*, *MPK3*) changed at 0.8 mg/ml. It means *MTT2/4* gene was more sensitive to toxicity of koumine than other selected genes. MTTs are effective at decreasing free radicals and the gene promoter is most likely very sensitive to the stress as it is in other eukaryotes [51]. *CYP1* is increased by oxidative stress in fish hepatic cell lines and zebrafish tissue [52, 53]. In our study, we found that the level of *CYP1* expression decreased at high koumine levels and was consistent with the previous experiments. *HSP70* is ubiquitously expressed heat shock protein that protect cells from stress. For instance, *HSP70* is overexpressed in response to oxidative stress in the pre-eclamptic placenta [27]. *HSP70* also protects human neuroblastoma cells from apoptosis and oxidative stress [54]. Therefore, oxidative stress can increase the expression level of *HSP70*. our study shows the same trend and *HSP70* levels increased significantly at 0.8 mg/mL koumine.

*MPK1* and *MPK3* are critical components of MAP kinase signaling and their expressions were related to oxidative stress [28, 29]. Studies have shown that oxidative stress may be regulated by MAPK pathway [55, 56, 57]. In our study, *MPK1* and *MPK3* increased significantly at 0.8 mg/mL koumine and suggested that koumine may involve oxidative stress through the MAPK pathway. But the specific mechanism of this action still needs further study. In addition, *ATG7* gene plays an important role in autophagy signal transduction and oxidative stress [30]. *ATG7* is involved in oxidative stress-induced autophagy in mouse cardiac progenitor cells [58]. In our study, *ATG7* expression was significantly increased at high concentration of koumine indicating involvement with oxidative stress-induced autophagy cells.

In summary, we preliminary investigated the toxicity of koumine in *T*. *thermophila* and found that koumine can inhibit *T*. *thermophila* growth. This is the first report that koumine exposure to T. *thermophila* causes oxidative stress leading to apoptosis, DNA damage and mitophagy. These toxic effects may be through MAP kinase pathway. Therefore, this study provides data for the future koumine toxicity studies in other pathogenic eukaryotic microorganisms and help us understand its toxicity more clearly.

## Supporting information

S1 File Supporting information used in the manuscript. Tables A and B and Figs A, B, C, D, E, F, and G in S1 File.
(DOC)
