# Supplementary material for: Induction of oxidative stress, apoptosis and DNA damage by koumine in Tetrahymena thermophila
Source: PLoS One. 2019 Feb 12;14(2):e0212231. doi: 10.1371/journal.pone.0212231 (PMC6372211; doi:10.1371/journal.pone.0212231)
Supplement: S1 File — Tables A and B and Figs A, B, C, D, E, F, and G in S1 File. (DOC) [file pone.0212231.s001.doc]

**Induction of oxidative stress, apoptosis and DNA damage by koumine in *Tetrahymena thermophila***

Qiao Ye^1, 2^ , Chaonan Zhang^1, 2^, Zhenlu Wang^1, 2^, Yongyong Feng^1, 2^, Aiguo Zhou^1, 2^, Shaolin Xie^1, 2^, Qiong Xiang^3^, Enfeng Song^3^ and Jixing Zou^1, 2^

^1^Healthy Aquaculture Laboratory, College of Marine Sciences, South China Agricultural University , Guangzhou, Guangdong, China.

^2^Joint Laboratory of Guangdong Province and Hong Kong Region on Marine Bioresource Conservation and Exploitation, College of Marine Sciences, South China Agricultural University, Guangzhou, Guangdong, China.

^3^Department of Traditional Chinese Medicine, Renmin Hospital of Wuhan University, Wuhan, Hubei, China.

Corresponding author

E-mail: [zoujixing@scau.edu.cn](mailto:zoujixing@scau.edu.cn) (J. X. Zou).

**Supporting information used in the manuscript.**

**Table A. Primers sequences used for RT-qPCR analysis.**

| Primer Name | Sequence (5' to 3') | Tm (℃) | Purpose |
| --- | --- | --- | --- |
| MTT2/4 | F: ATCCCTGCTCTTGTAATCCC | 54.1 | RT-qPCR |
|  | R: AGTTGGAAGTAGAACCGCA |  |  |
| HSP70 | F:TGAGAATCATCAACGAACCCAC | 54.9 | RT-qPCR |
|  | R:CGAAGATACCGTCATCAAGAGTAA |  |  |
| CYP1 | F: AGTGATTATTGCCTCATTCTTTGG | 51.2 | RT-qPCR |
|  | R: TGTTCTTCAGTAACCCCTAATTCG |  |  |
| MPK1 | F:ATCCGAAAGCAAATCCACT | 52.1 | RT-qPCR |
|  | R:TCAGGTTCTTCATCAGGGT |  |  |
| MPK3 | F:AAGGGTTTGAAGTATCTCC | 51.2 | RT-qPCR |
|  | R:CAGCCTTTGAGTAAACATG |  |  |
| ATG7 | F:CAGTTACTCGTCCTGGTT | 51 | RT-qPCR |
|  | R:CTCTGCCATACATCACTCT |  |  |
| 18S rRNA | F: CCTGGGAAGGTACGGGTAAT | 53.7 | RT-qPCR |
|  | R: AAGGTTCACCAGACCATTCG |  |  |

**Table B. *T. thermophila* cell densities at different times and koumine concentrations.**

| processing time | Blank control | Negative control | 0.05 mg/mL | 0.1 mg/mL | 0.2 mg/mL | 0.4 mg/mL | 0.8 mg/mL |
| --- | --- | --- | --- | --- | --- | --- | --- |
|  | Least-squares mean ± SEM (×10^5^ cells/mL) | | | | | | |
| 12h | 1.45±0.08^a^ | 1.41±0.06^a^ | 1.29±0.13^a^ | 1.18±0.11^b^ | 0.97±0.18^b^ | 0.88±0.13^b^ | 0.61±0.09^c^ |
| 24h | 3.16±0.28^a^ | 2.75±0.11^a^ | 2.47±0.24^a^ | 1.91±0.21^b^ | 1.66±0.18^c^ | 1.38±0.04^c^ | 0.91±0.16^d^ |
| 36h | 3.75±0.33^a^ | 3.54±0.36^a^ | 2.73±0.41^b^ | 2.36±0.17^b^ | 1.85±0.16^c^ | 1.63±0.09^c^ | 0.53±0.15^d^ |
| 48h | 5.03±0.28^a^ | 4.78±0.25^a^ | 4.06±1.02^a^ | 2.82±0.13^b^ | 2.67±0.12^b^ | 1.76±0.07^c^ | 0.41±0.16^d^ |
| 60h | 5.39±0.37^a^ | 4.91±0.07^a^ | 4.18±1.09^b^ | 2.64±0.06^c^ | 2.38±0.09^c^ | 1.69±0.06^d^ | 0.49±0.15^e^ |
| 72h | 5.52±0.49^a^ | 5.11±0.17^a^ | 4.37±0.96^a^ | 2.75±0.1^b^ | 2.44±0.13^b^ | 1.35±0.13^c^ | 0.3±0.19^d^ |

Data are summarized as means ±SEM

Values within a row with no common superscript differ significantly (*P* < 0.05) or are highly significant (*P* < 0.01).


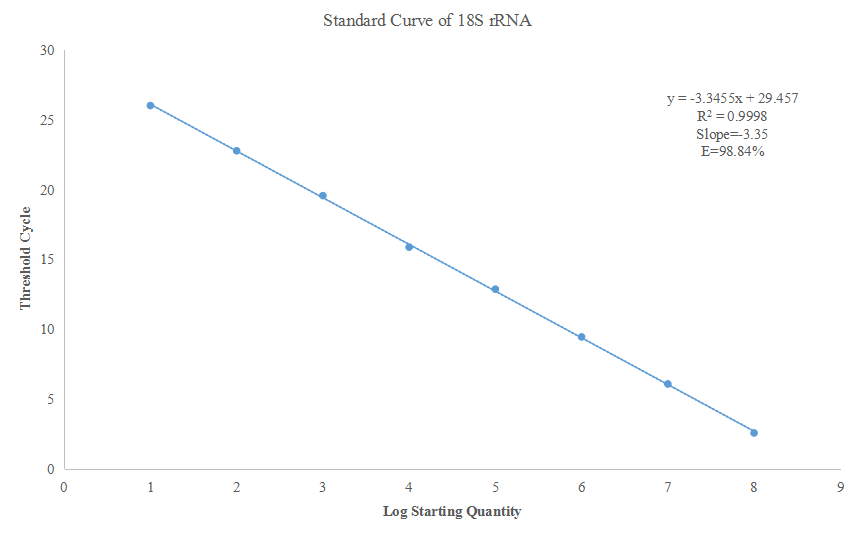


**Fig A. Standard curve of *18S rRNA* using in the RT-qPCR.** Correlation coefficient (R2), slope (S) and PCR efficiency (E) were 0.9998, 3.35 and 98.84, respectively.


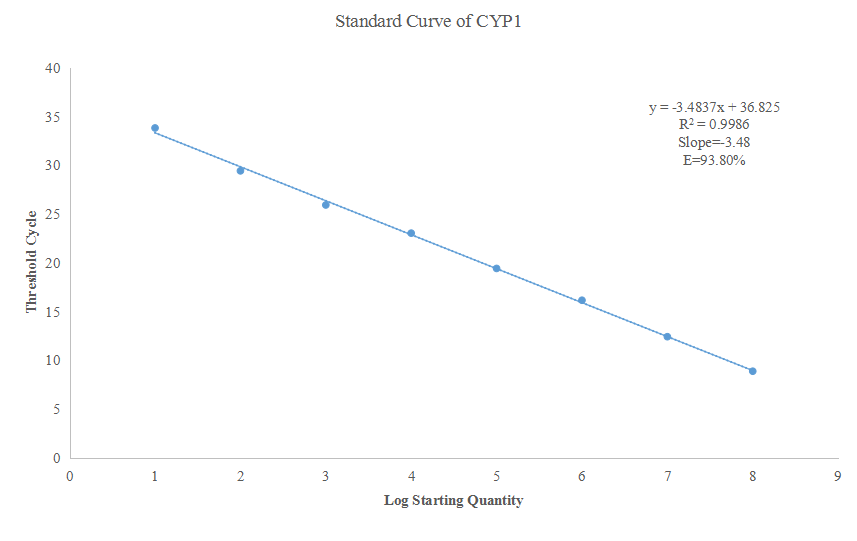


**Fig B**. ***CYP1* RT-qPCR standard curve.** R^2^=0.9986, S=3.48, and E=93.80%.


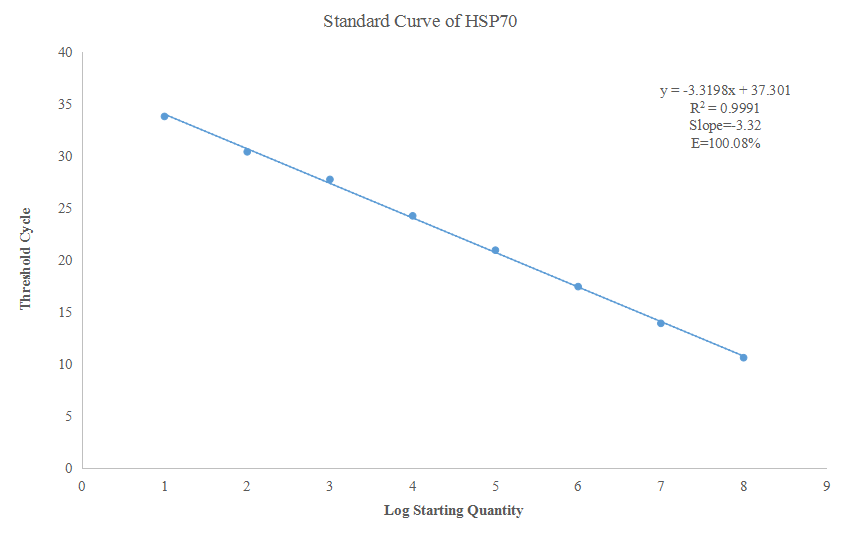


**Fig C. *HSP70* RT-qPCR standard curve.** R^2^=0.9991, S=3.32, and E=100.08%.


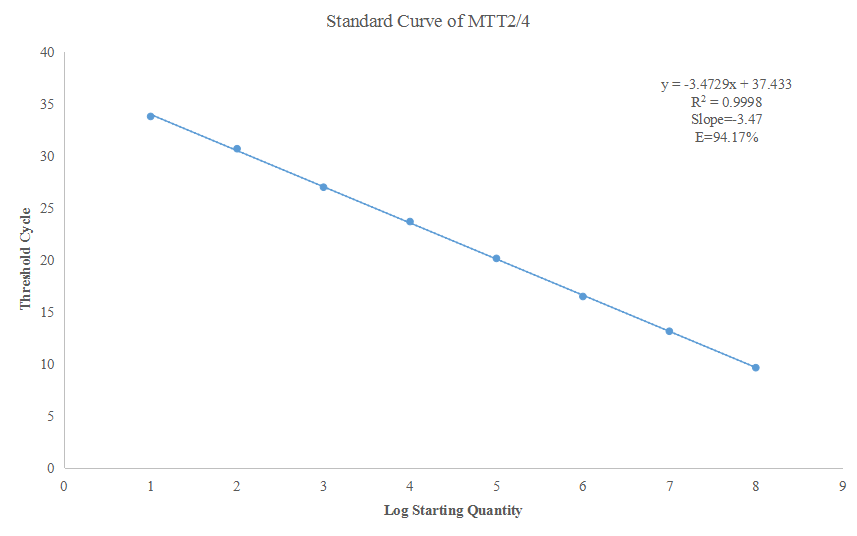


**Fig D. *MTT2/4* RT-qPCR standard curve.** R^2^=0.9998, S=3.47, and E=94.17%.


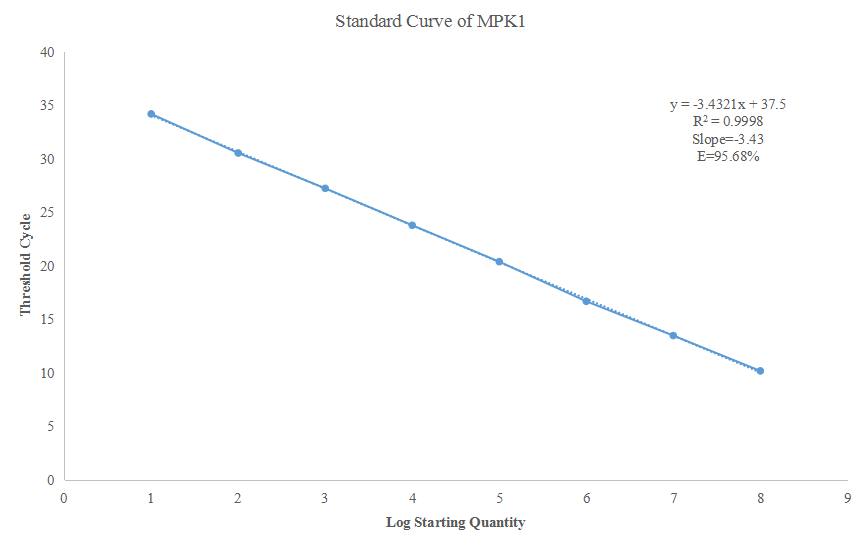


**Fig E. *MPK1* RT-qPCR standard curve**. R^2^=0.9998, S=3.43, and E=95.68%.


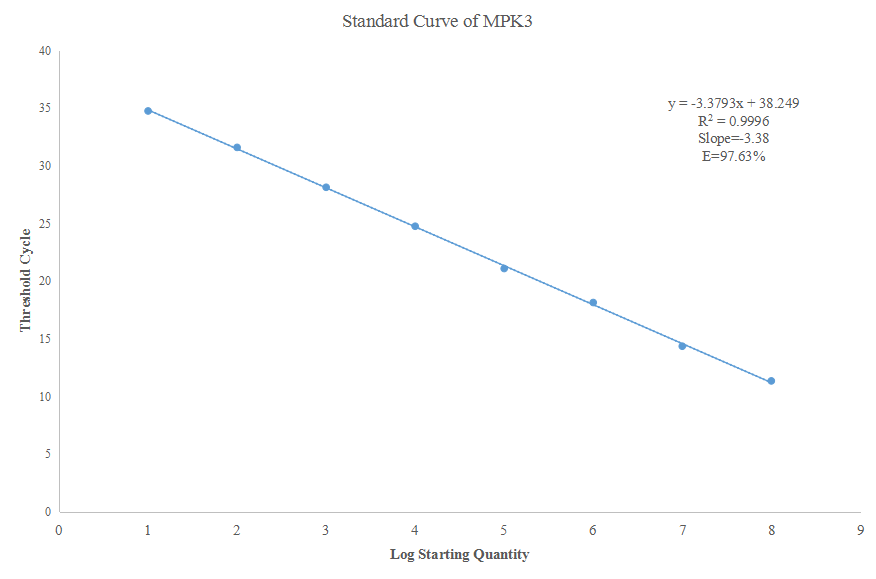


**Fig F. *MPK3* RT-qPCR standard curve.** R^2^=0.9996, S=3.38, and E=97.63%.


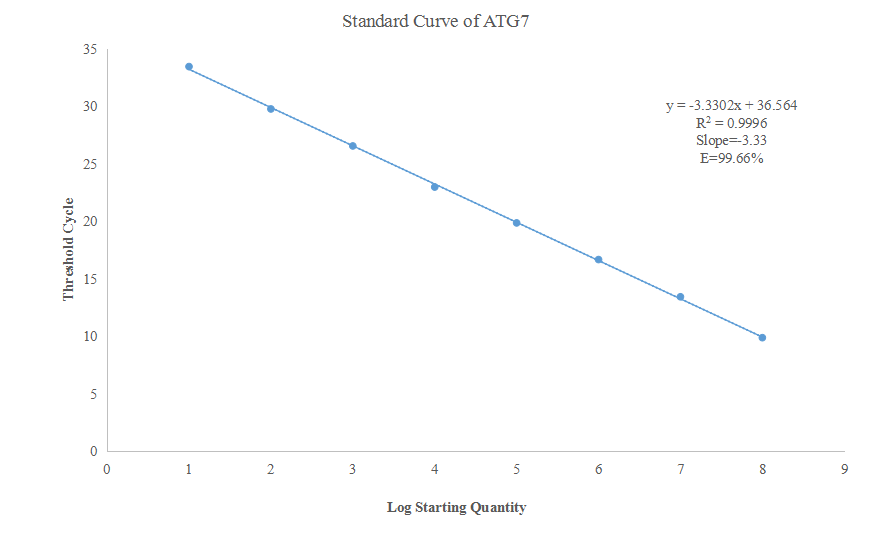


**Fig G. *ATG7* RT-qPCR standard curve**. R^2^=0.9996, S=3.33, and E=99.66%.
